# Antisocial and impulsive personality traits are linked to individual differences in somatosensory maps of emotion

**DOI:** 10.1038/s41598-023-27880-1

**Published:** 2023-01-12

**Authors:** Soren Wainio-Theberge, Jorge L. Armony

**Affiliations:** 1 Douglas Mental Health University Institute, 6875 LaSalle boulevard, Verdun, QC H4H 1R3, Canada; 2 Integrated Program in Neuroscience, McGill University, Montreal, QC Canada; 3 Department of Psychiatry, McGill University, Montreal, QC Canada

**Keywords:** Psychology, Emotion, Sensorimotor processing, Somatosensory system

## Abstract

Somatosensory experience is an important component of emotion, playing a prominent role in many traditional emotion theories. Nonetheless, and despite the extensive literature on the influence of individual differences in emotional processing, the relation between personality traits and emotion-related somatosensation has received little attention. Here, we addressed this question in a large sample of healthy individuals through the “bodily maps of emotion” behavioural paradigm, in which participants indicated the location and extent of their body sensations for the 6 basic and 4 additional social emotions (contempt, envy, pride, shame). We found that emotional somatosensation in specific body areas, including the heart, the stomach, and the head, was related to specific personality factors, particularly antisocial attitudes and impulsivity. Moreover, the similarity of individual participants’ maps to the group-average was likewise negatively correlated with antisocial tendencies. Overall, our results suggest that differences in individuals’ sensitivity to somatosensation from different body areas, as well as the typicality of their topographical patterns, may partly underlie variation in higher-order social and affective traits.

## Introduction

Emotions are thought to reflect states of whole-body coordination, orchestrated by the brain to achieve evolutionarily salient goals. This physical response involves changes in the autonomic nervous system^1^, including changes in heart rate, blood pressure^2,3^, skin temperature^4^, and respiration^5^, as well as predisposing whole-body movements and postures^6^. The somatosensory feedback from these processes has a prominent role in many theories of emotion, including William James’ early model^7^, which proposed that the emotional experience emerges from the perception of physiological events automatically triggered by biologically-relevant (e.g., threat) stimuli. Other, later models added a cognitive component for the interpretation of these physiological processes in terms of specific emotional experiences^8^. Supporting these theories, recent studies using neuroimaging and brain stimulation techniques have found that somatosensory processes are key to processing emotions in both the self^9^ and others^10,11^.

Notably, there seems to be a general agreement in (some) characteristic bodily sensations of emotions, as evidenced by their representation in common idioms in many languages, such as the feelings of “butterflies in one’s stomach” or the feeling of one’s “heart sinking”. More recently, a behavioural method has been developed to directly and quantitatively measure individuals’ emotion-related bodily sensations. In the “bodily maps of emotion” paradigm employed by Nummenmaa et al.^12^, participants “paint” areas of an on-screen manikin in which they feel increases or decreases in somatic sensation in response to particular emotions. This procedure reveals that different emotions have distinct patterns of associated somatosensation. For example, anger features heightened sensation (*activation*) in the chest, arms, and head, while sadness involves activation in the torso and reduced sensation (*deactivation*) in the limbs^12^. Interestingly, the spatial patterns of bodily maps of the different emotions converge throughout development towards those drawn by adults, which could reflect increasing accuracy and awareness of emotion-related bodily sensations with age^13^.

Importantly, these *bodily sensation maps* (BSMs) have been shown to be consistent across different methods of emotion induction (e.g., recall, emotional faces and movies^12,14^), as well as concordant across cultures and between men and women^15^. Nonetheless, significant group differences in BSMs have also been reported, especially in clinical populations. For instance, Palser et al. found that BSMs drawn by children with autism spectrum disorders were less differentiated than those drawn by typically-developing children^16^, whereas Torregrossa et al. found a similar pattern when comparing patients with schizophrenia to healthy controls^17^. Finally, Lyons et al. reported that BSMs of nonmedicated depressed individuals showed less overall activation compared to those drawn by healthy controls^18^. Taken together, these studies suggest that bodily maps of emotion capture variation in somatosensory processes as a function of disease. Given that disease states often reflect extreme versions of traits that exist in the general population^19,20^, it could be expected that variability in emotional somatosensation may also be present in healthy individuals, and related to personality factors.

Particularly relevant candidate traits are those that have been shown to modulate emotional sensations. For instance, individuals high in empathy tend to experience emotions as more differentiated^21^, as do individuals high in interoceptive sensitivity^22^. This ability to experience fine-grained distinctions between emotional experiences is referred to as emotional granularity^23^ and is predictive of resilience to psychiatric disease, as well as serving as a protective factor against aggressive tendencies and alcohol abuse^24^. Similarly, it is known that impulsivity is associated with impaired emotion regulation abilities^25^, and that impulsive individuals are more inclined to act on strong positive and negative emotions^26^.

Thus, the aim of this study was to employ the bodily maps of emotion paradigm to investigate individual differences in emotion-related somatosensation as a function of personality. Specifically, we conducted a data-driven investigation to determine if and how major personality traits—such as positive and negative affect, impulsivity, pro- and anti-social attitudes and interoceptive ability—were related to individual variations in emotional somatosensation. Based on previous research using the BSM paradigm in healthy and clinical populations^12,13,17^, we examined two main aspects of emotional somatosensation in relation to personality. First, we considered somatosensation in different body areas, assessed using principal component analyses of participants’ maps. Second, inspired by the emotional granularity research^24^, we used a linear discriminant analysis classifier as a proxy for the between-emotion and between-subject distinctiveness of somatosensory maps. To further assess the relative contributions of these two factors to the observed relation between certain personality traits and classification confidence, we performed a post-hoc analysis in which we calculated the cosine distance between participants’ maps and the group average and correlated it with those personality components. In brief, we found widespread relationships between personality variables and features of emotion-related somatosensation, including several features of BSMs predicting antisocial tendencies, interoception, impulsivity, and negative affect. These results suggest that personality is an embodied phenomenon, with individual differences in somatosensory processes having upstream effects on higher-level cognition and personality.

## Methods

### Participants

A total of 362 volunteers (mean age = 20.9 years, SD = 2.0; 54 males) were recruited as part of a broader online study on body posture, emotional perception, and personality. Participants were recruited from the general public using social media advertisements (n = 63) and from the McGill University Department of Psychology extra credit participant pool (n = 299). The study was approved by the McGill University Faculty of Medicine Institutional Review Board (IRB# A00-B62-21A) and written informed consent was obtained from all participants. They were either compensated with CAD 15$ for their participation or received course credit. The study procedures were carried out in accordance with the Declaration of Helsinki.

### Experimental procedure

The study consisted of four 15-min experimental modules run on a JATOS server^27^, hosted by the International Laboratory for Brain, Music, and Sound Research (BRAMS), using the jsPsych library^28^. One module consisted of an unrelated behavioral task, not reported here. Two other modules consisted of personality questionnaires (described in *Personality measures*, below).

The final module consisted of the bodily maps of emotion task from Nummenmaa et al.^12,14^. Participants were presented with emotion words and two silhouettes of bodies and asked to paint, using a mouse, areas of their body whose activity they felt increasing or getting stronger on one silhouette (referred to as “activation” in the text; coloured in red), and areas whose activity they felt decreasing or getting weaker on the other (referred to as “deactivation”; coloured in blue), when experiencing that emotion. “Paint” was added continuously, such that increasing the time spent over a given region increased the opacity of colour over that region. Further details of the procedure are described in Nummenmaa et al.^12^. Participants created body sensation maps (BSMs) for the six basic emotions (anger, fear, sadness, happiness, disgust, surprise) as well as four social emotions (pride, shame, contempt, and envy) of the 14 reported in Nummenmaa et al., to reduce the duration of the experiment.

Finally, participants answered five questions about their experience of each emotion, drawn from Nummenmaa et al.^14^: (i) how much do you feel this emotion in your body, (ii) how much do you feel this emotion in your mind, ((iii) how pleasant does this emotion feel, (iv) how much control do you feel you have over this emotion, and (v) how frequently do you experience this emotion. Participants answered these questions on 5-point Likert scales immediately following completion of each bodily map.

### Personality measures

A battery of personality questionnaires was administered in two modules (personality modules 1 and 2). Personality module 1 included the Barratt Impulsivity Scale (BIS-11; Patton et al.^29^), the Big Five Inventory (BFI; John et al.^30^), the Interpersonal Reactivity Index (IRI; Davis^31^), the Multidimensional Assessment of Interoceptive Abilities Version 2 (MAIA-2; Mehling et al.^32^), the Levenson Self-Report Psychopathy Scale^33^, the Social Dominance Orientation scale (SDO; Pratto et al.^34^), and the Life Orientation Test Revised (LOT-R; Scheier et al.^35^). Personality module 2 consisted of Positive and Negative Affect Schedule—Expanded (PANAS-X; Watson and Clark^36^), the Spielberger State-Trait Personality Inventory (STPI; Spielberger and Reheiser^37^), the State-Trait Anger Expression Inventory (STAXI; Spielberger et al.^38^), the Toronto Alexithymia Scale (TAS; Bagby et al.^39^), the Cognitive Emotion Regulation Questionnaire (CERQ; Garnefski and Kraaij^40^), and the Psychological Well-Being Scale (PWB; Ryff^41^). These scales were chosen due to their relevance to trait emotionality and emotion processing in healthy populations. Although state emotion in the PANAS-X and STPI was also collected (for the behavioral task), only trait emotionality was considered here, as we were interested in the relation between body sensation and stable personality traits.

### Data preprocessing and reduction

We screened the body map and personality data for random responding and non-compliance as follows: For the body map data, we visually inspected each map, removing participants if they had drawn clear symbols (e.g. smiley faces, hearts), marked only single dots rather than colouring in areas, or circled regions instead of colouring them in (these behaviours indicated a lack of understanding of, or willingness to follow, the instructions). Participants were also removed if they failed to colour any area for 3 or more emotions (i.e. more than 25% of body maps). For the personality data, a multivariate outlier detection procedure was used to screen for random responders^42^. Since there were more questions in the personality battery than participants (536 vs. 362), we conducted the Hadi procedure 1000 times on random subsets of 50% of the questions in the personality battery. Participants who were marked as outliers at the 5% significance level in more than 10% of these random splits were removed.

### Data reduction of personality and body maps: principal component analyses and correlation

To reduce the personality and BSM data, we performed principal component analyses on each, separately, using the same approach for each data type. For BSM data, pixels within the body silhouette were taken as variables (50,364 pixels total) and individual maps were taken as observations: thus, there were 10 observations per participant, corresponding to that participant’s map of each emotion. For the personality data, each personality scale was its own variable, and subjects were observations. The number of components retained in each PCA was determined using a permutation test procedure. In order to account for heterogeneous noise which may be present in both datasets (particularly the body maps), we used the procedure from Hong et al.^43^. Briefly, the eigenvalues obtained from the PCA were compared with the eigenvalues obtained from 1,000 permutations of the data created by applying a matrix of random sign-flips, rather than shuffling the data as in a standard permutation test. For both the personality and body map PCA analyses, components whose eigenvalue exceeded the 95th percentile of this permutation distribution were retained. To improve interpretability, factor rotation was then performed on the retained components using Quartimax rotation. Significant loadings for each retained component were then calculated using bootstrapping^44^: 1,000 bootstrap resamplings of the data were computed, and bootstrapped loading z-scores were created by dividing each loading by its bootstrap standard deviation.

Our first analyses then consisted of correlating the components obtained from the personality and body maps PCAs with each other using Spearman’s rank correlation. As over 50 components were obtained for the bodily maps using the permutation test, a smaller number of components (4) was selected for the correlation analyses by visual inspection of the eigenvalue plots. Multiple comparisons were corrected for using False Discovery Rate correction^45^.

### Emotional granularity: linear discriminant analysis classification and analyses of differentiation and representativeness

We trained a linear discriminant analysis (LDA) classifier to classify emotions from participants’ BSMs, and related the confidence of this classification to personality factors. Linear discriminant analysis classifiers are a family of classifiers which attempt to find a linear decision boundary between classes in a multidimensional space. Here, the data used in the LDA classifier consisted of the emotion- and subject-specific scores of the significant BSM principal components obtained in PCA described above. These data were then used to predict the emotion category of each map. The LDA classifier was trained using the default settings of the *fitcdiscr* function in MATLAB R2020a, with the prior distributions for each class taken as the empirical frequencies within each class. Significance of the classifier was assessed using standard parametric statistics (χ^2^ test) and a multiple cross-validation procedure: 1000 random splits of the data were created, and for each split the classifier was trained on one half of the data and tested on the other half. A* p* value was generated by calculating the percentile of 10% (chance level) within the distribution of test-set accuracies.

As our subjects did not belong to a priori different groups (e.g., clinical and healthy, as in Torregrossa et al.^17^), to relate classifier accuracy to personality we used the posterior probabilities generated by the LDA as an index of the confidence of the classifier in classifying any given map^46^. These classifier confidence scores were then correlated with the personality PCA components obtained above. Specifically, we calculated the posterior probability that each map would be correctly classified as the emotion label for which it was drawn; we then averaged these probabilities for each subject to obtain a subject-level classification confidence score. This classification confidence score was then correlated with the personality PCA components using Spearman’s rank correlation.

In order to accurately discriminate data from different classes, classifiers such as linear discriminant analysis need not only high inter-class variability, but low within-class variability^47,48^. These properties can be related to the *differentiation* and *representativeness* of participants’ maps, respectively. Differentiation refers to how distinguishable participants’ maps are between emotions; that is, does a participant paint the same sensation map for every emotion, or are emotions differentiated in the magnitude or location of their sensations? Representativeness, on the other hand, refers to the degree to which an individual BSM resembles the group-average map: that is, does a participant’s map of a given emotion resemble the group average, or is it instead idiosyncratic or random?

We sought to assess the extent to which any correlation between personality and classification accuracy was driven by each of these properties. In order to determine to what extent differences in classifier accuracy were driven by differentiation and/or representativeness in BSMs, we operationalized these concepts using cosine-distance-based metrics. Differentiation was operationalized as the within-subject average pairwise cosine distance between emotions in the body-map PCA space (i.e., higher values represented larger differentiation between emotions). Representativeness, on the other hand, was implemented as a participant’s average pairwise cosine similarity between their maps and the group average for each emotion (i.e., higher values corresponded more representative BSMs).

We used cosine similarity/distance as we were interested in pattern similarity across emotions/subjects, regardless of the magnitudes (pixel values). This approach, commonly used in machine learning research for evaluating the similarity of images^49^, was chosen in order to minimize individual differences in the interpretation of instructions and the amount of painting, and is and equivalent to measures used previously to compute similarities in BSMs across groups^13^.

## Results

### Correlation between personality and bodily sensation maps

**Figure 1:**
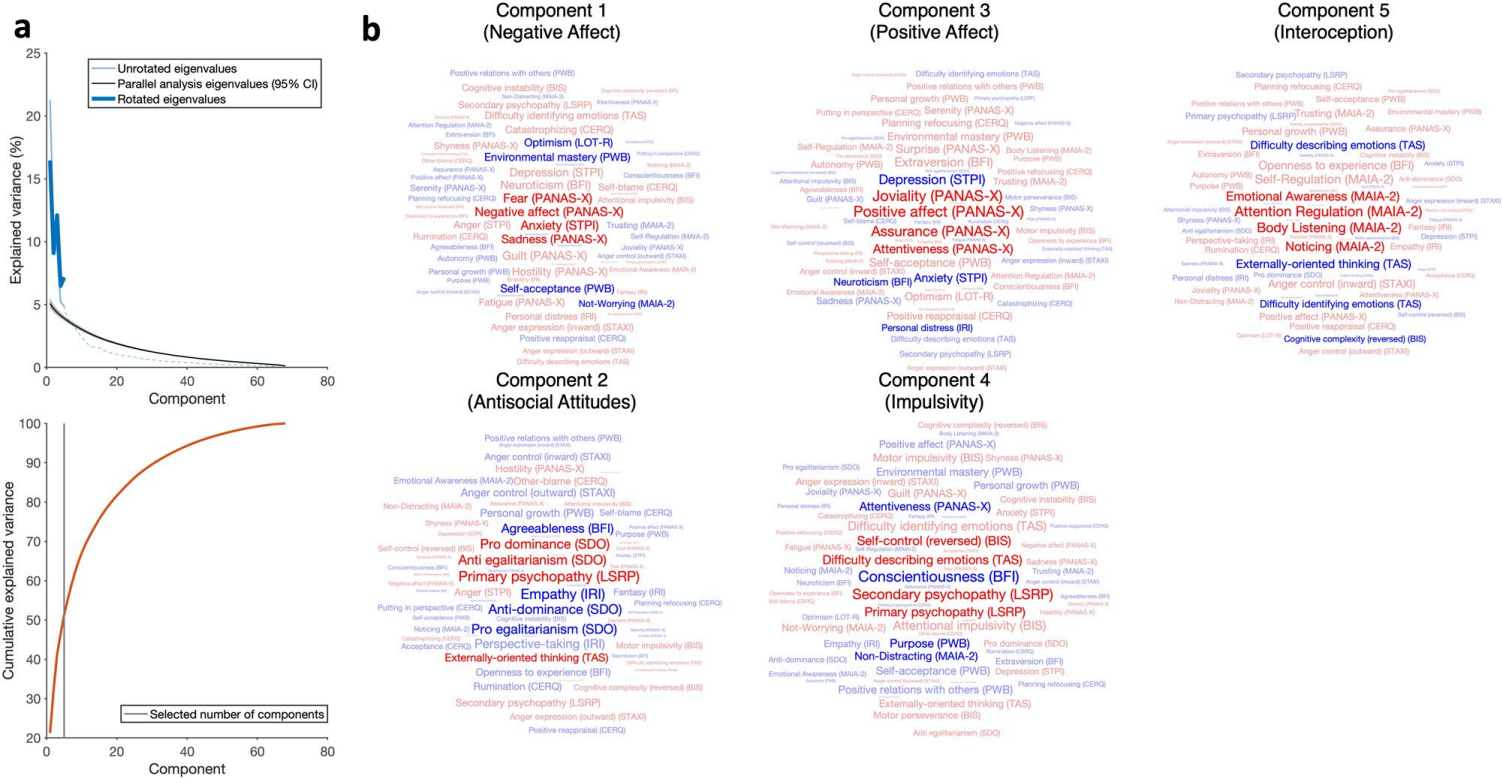
Latent dimensions of personality uncovered using PCA and used in the linear modelling procedure. (**a**) Eigenvalue plot showing the distribution of eigenvalues by component. Light blue line shows the unrotated eigenvalues; the dashed part reflects eigenvalues not significant following the permutation test. Black line shows the eigenvalues from the permutation test and the 95% confidence interval of these (grey shading). Dark blue shows the eigenvalues of the components following quartimax rotation. (**b**) Loadings of all significant components (determined by the permutation test). The size of the word corresponds to the magnitude of the loading, and red words reflect positive loadings while blue words reflect negative loadings.

After removal of participants based on visual inspection of bodily maps and the multivariate outlier-detection procedure for personality (see “Methods”), the final dataset consisted of 228 participants (mean age 20.49, SD = 1.58; 32 males). The principal components analysis on the personality data, designed to reduce the number of comparisons and yield interpretable findings from our large battery of personality scales, yielded 5 statistically significant components (Fig. 1): components loaded mainly on (1) negative components of PANAS, anxiety, and negatively on optimism, environmental mastery and self-acceptance (henceforth named *Negative Affect*); (2) psychopathy and social dominance and negatively on empathy (*Antisocial Attitudes*); (3) positive affect and negatively on anxiety and depression (*Positive Affect*); (4) impulsivity and secondary psychopathy and negatively on conscientiousness and attentiveness (*Impulsivity*); and (5) positive on interoceptive abilities and emotional awareness and negative on difficulty to describe and identify emotions (*Interoception*). Full component loadings with bootstrap confidence intervals are reported in the supplementary materials (Supplementary Fig. S1).

**Figure 2:**
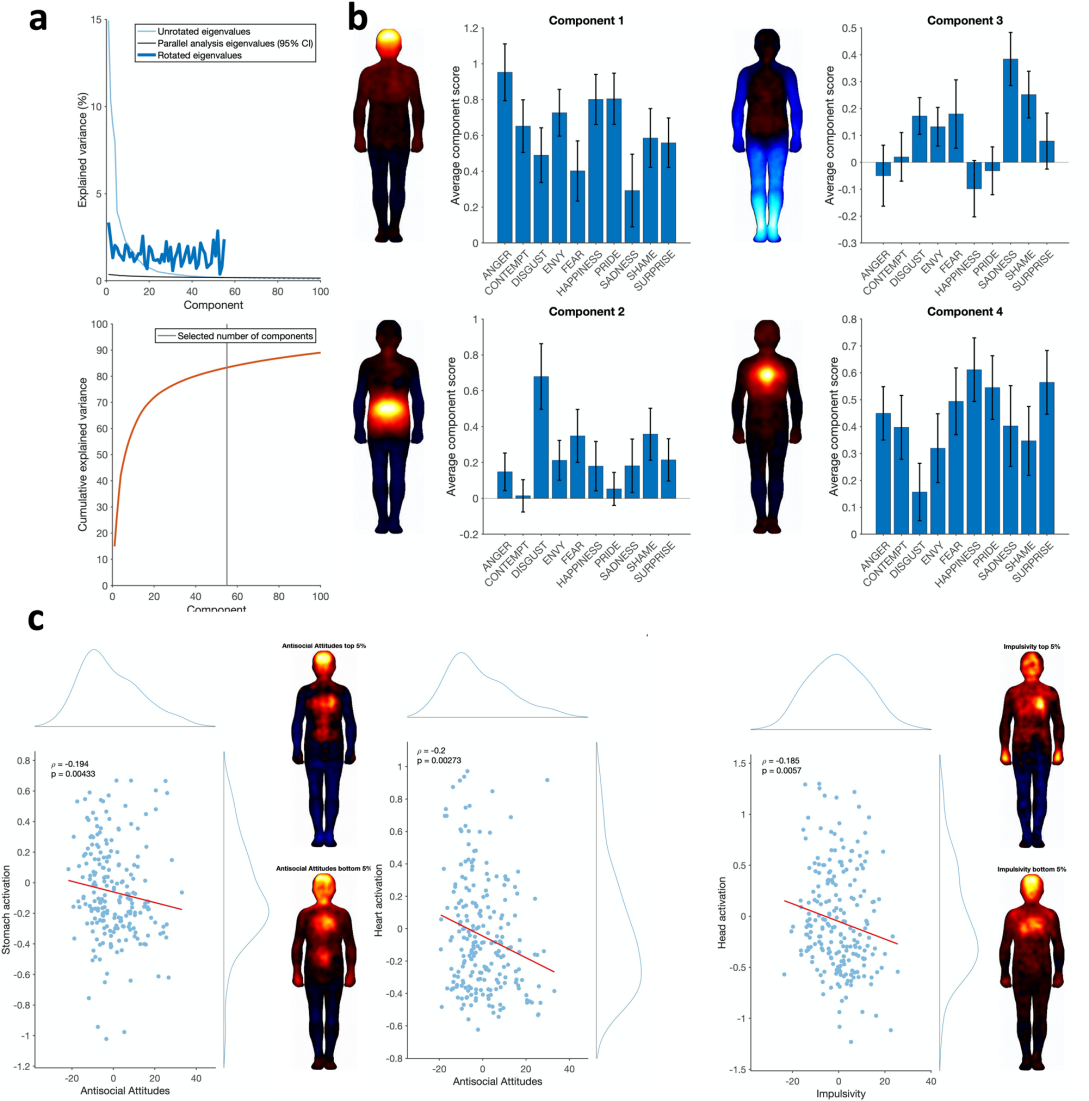
Results of the PCA of the body map data and correlation of BSM and personality PCA components. (**a**) Eigenvalue plot for the BSM PCA, as in Fig. 1a. (**b**) Loadings and averaged component scores for the top 4 components. Silhouette plots show the loadings of the rotated component. Bar plots show the scores of the component, averaged over subjects within each emotion; error bars indicate standard error. These bars indicate the magnitude at which the component is activated in each emotion. (**c**) Correlation analysis of body map and personality PCA components. Spearman’s ρ and its corresponding *p* value are indicated. Histograms show the distribution of each variable. Silhouette plots show averaged BSMs from the extremes of the personality distribution (top and bottom 5% of each personality PCA component), averaged across emotions.

For the bodily sensation maps, the parallel analysis yielded 55 components which were significant above the permutation threshold (Fig. 2a, top). The first 4 of these components are displayed in Fig. 2b. The first component reflected mainly activation in the head and upper chest (referred to as *Head Activation*), and was strongly represented in anger, happiness and pride. The second component reflected activation in the stomach area (*Stomach Activation*), and was mainly present in disgust and, to a lesser degree, in fear and shame. The third component reflected deactivation in the limbs, particularly the legs and feet (*Legs Deactivation*); this component was particularly present in sadness and shame, as well as fear and disgust. Finally, the fourth component represented activation in the heart (*Heart Activation*) and was present in all emotions, but particularly strongly in happiness, pride, and surprise.

To determine if there were significant relationships between specific aspects of emotional body sensations and personality, we correlated subjects’ emotion-averaged BSM and personality PCA components. Full results of these correlations are reported in Table 1. Only two correlations survived FDR correction for multiple comparisons, with a third being marginally significant: A negative correlation of *Antisocial Attitudes* with *Stomach Activation* (ρ = − 0.21, p = 0.002, p_FDR_ = 0.03) and with *Heart Activation* (ρ = − 0.19, *p* = 0.003, p_FDR_ = 0.03), and an almost-significant negative correlation of *Impulsivity* with *Head Activation* (ρ = − 0.17, *p* = 0.01, p_FDR_ = 0.06; Fig. 2c).

**Table 1:** Correlations of personality and body map principal components.

	Head activation	Stomach activation	Legs deactivation	Heart activation
Negative Affect	− 0.056 (0.40)	− 0.080 (0.23)	− 0.057 (0.39)	− 0.051 (0.44)
Antisocial Attitudes	− 0.091 (0.17)	**− 0.207 (0.002)**	− 0.018 (0.79)	**− 0.194 (0.003)**
Positive Affect	0.007 (0.92)	0.027 (0.69)	− 0.052 (0.43)	− 0.049 (0.46)
Impulsivity	**− 0.172 (0.01)**	− 0.054 (0.42)	0.005 (0.94)	− 0.115 (0.08)
Interoception	0.023 (0.73)	0.096 (0.15)	− 0.134 (0.043)	0.105 (0.12)

Spearman’s rank correlation coefficients are presented with uncorrected* p* values in brackets. Significant or marginal results after FDR-correction for multiple comparisons are highlighted in bold.

### Emotional granularity in bodily maps of emotion: classifier confidence

**Figure 3:**
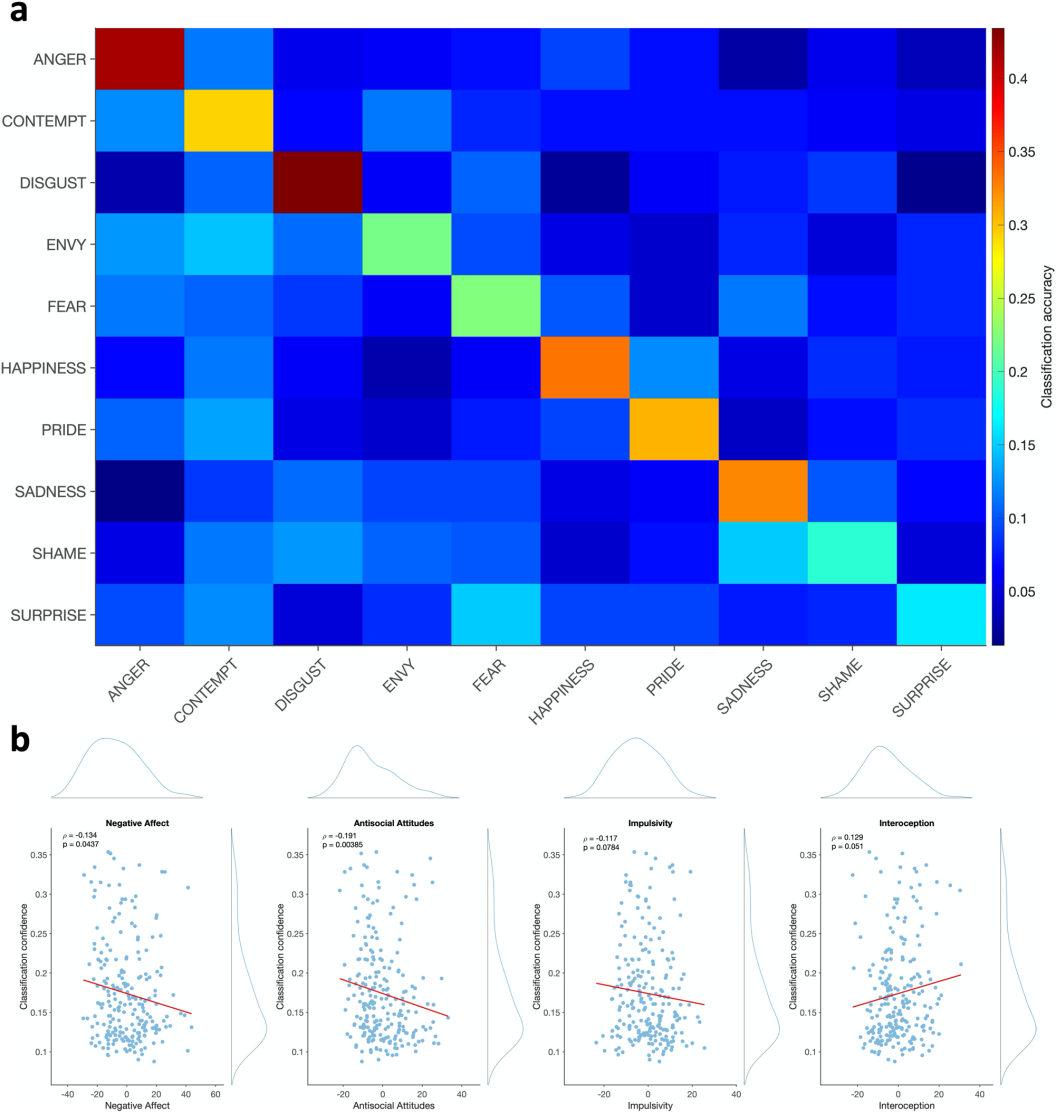
Linear discriminant analysis classifier confidence and its association with personality. (**a**) Confusion matrix for the linear discriminant analysis classifier predicting emotion label from participants’ BSM data. (**b**) Correlations of classifier confidence with personality PCA components. Classifier confidence was computed as the posterior probability that an emotion is accurately classified as its true label, averaged over emotions for each subject. Spearman’s ρ and uncorrected* p* values are indicated. Histograms show the distribution of each variable.

Next, we considered whether a BSM-based measure of emotional granularity (i.e., tendency to experience fine-grained distinctions between emotions^24^) might be related to personality features. Following Torregrossa et al.^17^, we considered the linear discriminant analysis BSM classifier accuracy as a proxy for emotion sensation differentiation. All 10 emotions were classified well above chance in an all-against-all classification scheme (average classification accuracy = 29%, chance level = 10%; χ^2^ = 1165.1, *p* < 0.001; Fig. 3a). The classifier was also significant following cross-validation (*p* = 0.01). We then correlated each subject’s average classification confidence (defined as the posterior probability that a given emotion would be classified as the correct emotion; see “Methods”) with that subject’s personality PCA components. We found that classification confidence was negatively correlated with *Antisocial Attitudes* (ρ = − 0.19, *p* = 0.004, p_FDR_ = 0.02) and *Negative Affect* (ρ = − 0.13, *p* = 0.04, p_FDR_ = 0.09; Fig. 3b), as well as marginally positively correlated with *Interoception* (ρ = 0.13, *p* = 0.05, p_FDR_ = 0.09).

### Emotion representativeness and differentiation: Cosine-based distance metrics

**Figure 4:**
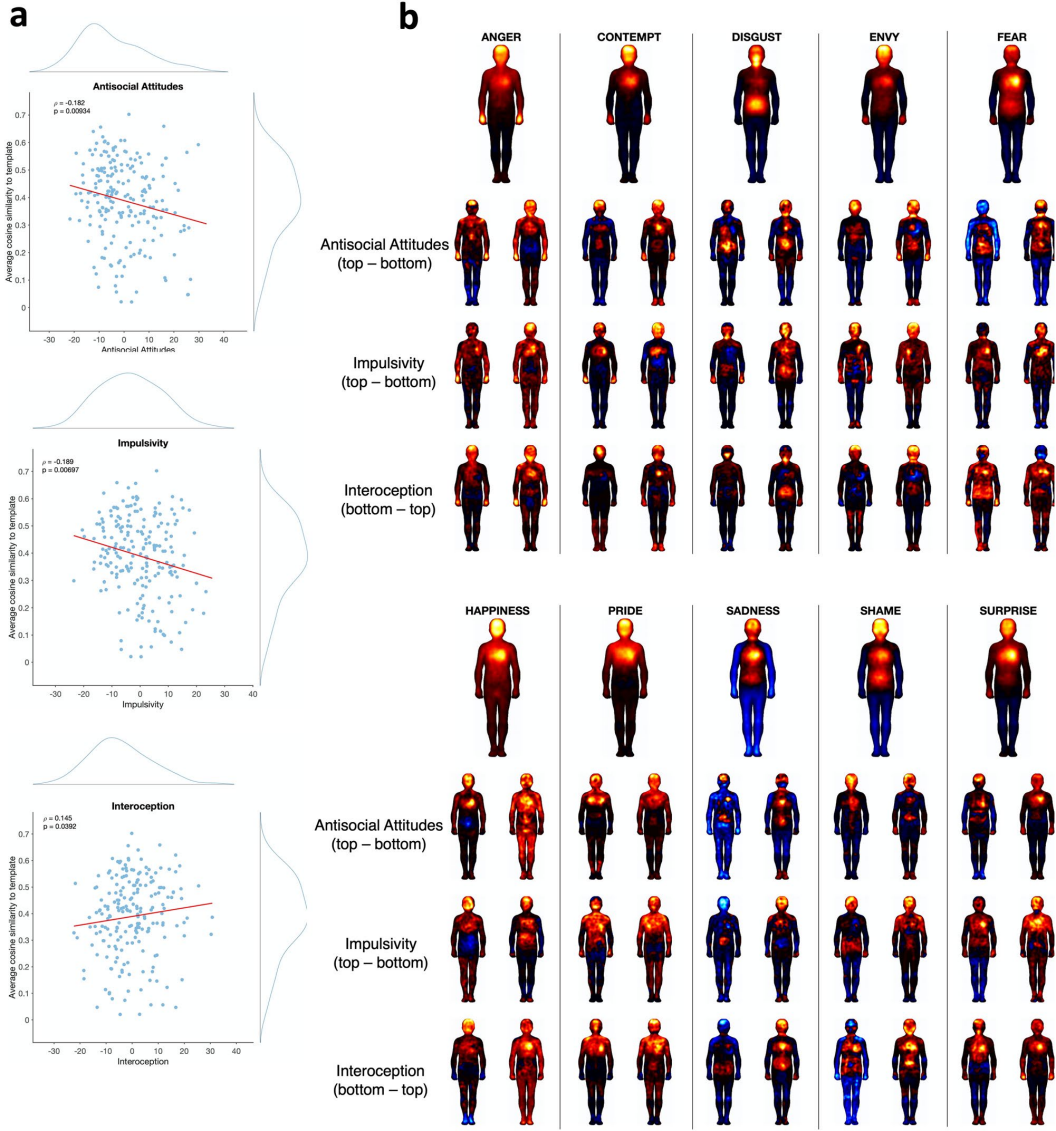
Relationship of body map representativeness and differentiation with personality PCA components. (**a**) Correlations of BSM representativeness with personality PCA components; Spearman’s ρ and the corresponding* p* value are plotted. Histograms show the distribution of each variable. (**b**) Average body maps for the bottom 5% and top 5% of each personality factor (empathy, interoception: bottom 5% on the left, top 5% on the right; impulsivity: top 5% on the left, bottom 5% on the right). Group average maps are shown above.

To assess the contributions of between-emotion distinctiveness and between-subject consistency to classification accuracy, we computed cosine-based distance scores in BSM PCA space (see “Methods” for details). Considering the representativeness of participants’ BSMs (i.e., similarity to the group mean), there were significant negative correlations with *Antisocial Attitudes* (ρ = − 0.18, *p* = 0.01, p_FDR_ = 0.03; Fig. 4) and *Impulsivity* (ρ = − 0.17, *p* = 0.01, p_FDR_ = 0.03), respectively, as well as a trend for a positive correlation with *Interoception* (ρ = 0.13, *p* = 0.06). In contrast, there were no significant correlations between emotion differentiation and personality factors, although there was a trend for a negative correlation with *Interoception* (ρ = − 0.14, *p* = 0.05).

*Antisocial Attitudes* also showed a trend towards a positive correlation with emotion differentiation (ρ = 0.12, *p* = 0.1); that is, higher *Antisocial Attitudes* scores were associated with lower BSM representativeness but higher emotion differentiation.

### Validation analyses: correlations with subjective reports of bodily/mental salience

As a validation of our analyses, we correlated the BSM PCA components with participants’ subjective reports of how much they felt each emotion in their body or their mind, respectively. We found that the *Heart Activation and Leg Deactivation* components of the BSMs were positively (ρ = 0.17, *p* = 0.009) and negatively (ρ = − 0.16, *p* = 0.01) correlated with body salience, respectively. Meanwhile, the *Head Activation* BSM component was significantly correlated with mental salience (ρ = 0.23, *p* = 0.0004).

## Discussion

**Figure 5:**
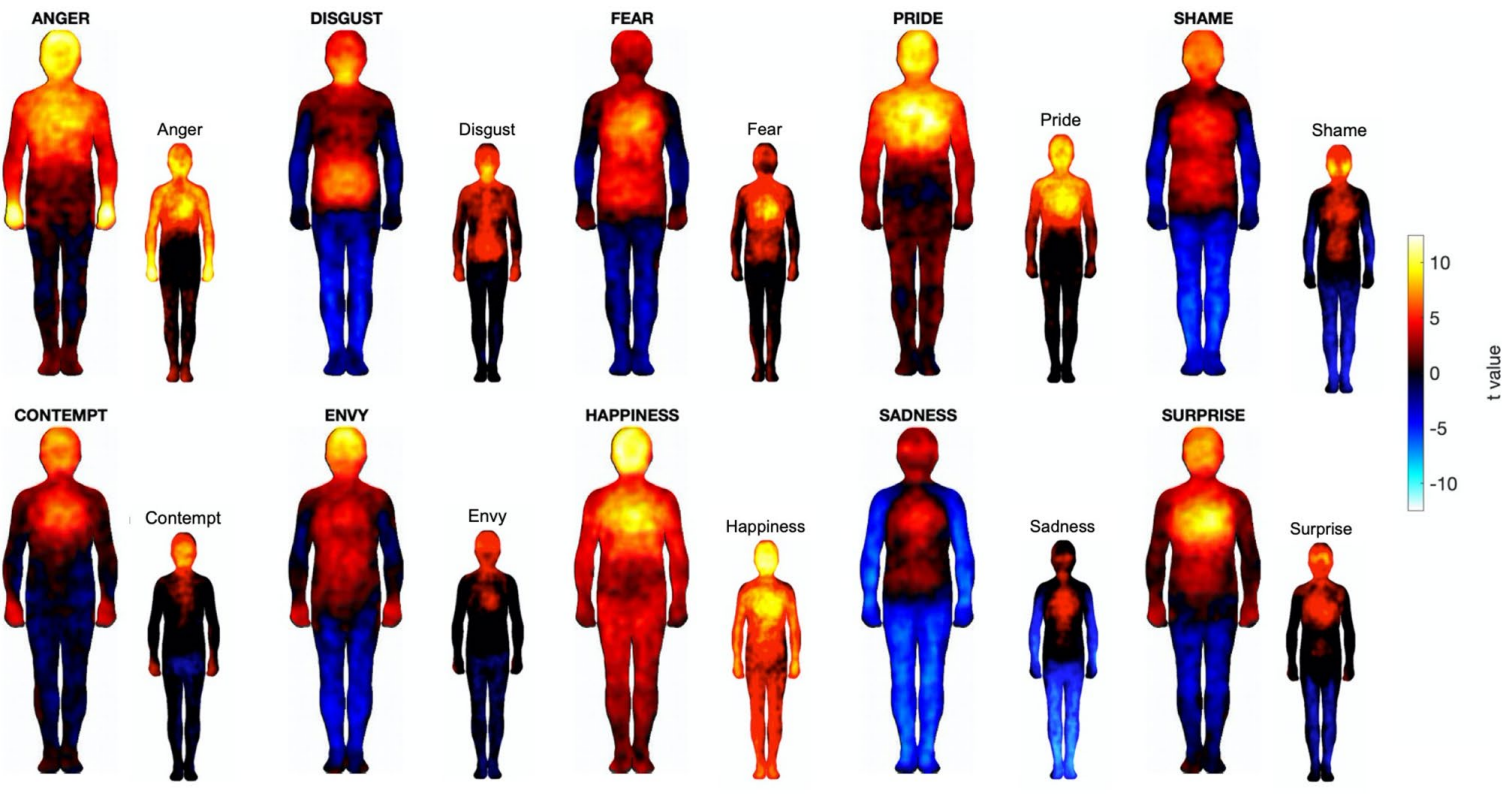
Group-level t maps for the bodily sensation maps analyzed in the present study. Large silhouettes show t-statistics from a t-test against zero calculated at each pixel in the body maps. Small silhouettes show the corresponding maps from Nummenmaa et al.^12^, to indicate correspondence.

Using a hypothesis-free, data-driven approach, we investigated the relation between individual differences in personality traits in healthy individuals and their representation of whole-body patterns of somatic sensation using the bodily sensation maps (BSM) paradigm^12^. In agreement with previous findings, we obtained consistent patterns of somatosensation which are stable across individuals. Indeed, the group-level bodily maps of emotions found in our study were highly similar to the ones originally presented by Nummenmaa and colleagues (Fig. 5). However, our findings also suggest that inter-individual variability exists in these maps, and that some of this variation is systematically related to personality (although the nature of our analyses does not allow for a determination of the causal direction of this relation). Specifically, we observed that several dimensions of participants’ emotion-related somatic sensations, including the amount of “activation” in the heart, viscera, and head, were associated with different personality features, namely antisocial tendencies, interoception and impulsivity. Moreover, we showed that patterns of variability in participants’ BSMs (including emotion differentiation and representativeness of the group-average) were also associated with personality. Overall, our results confirm previous work suggesting a role for somatosensation in emotion awareness and understanding, and extend it by providing possible mechanisms for some of the observed inter-individual variability and its relation to particular personality traits.

### Dimensions of emotional somatosensation and personality traits

We found that the bodily maps of emotion coloured by participants were well described by a lower-dimensional space composed of localized components representing different body areas. The first component was strongly localized to the upper head, and was implicated in most emotions; as such, it may index the somatic experience of cognition, as it correlated with the mental salience of emotions (see “Results”—“Validation Analyses”). Supporting this notion, recent work using the BSM paradigm has shown that “cognitive feelings”, such as the feelings associated with imagining, remembering, or being attentive, also have somatic components, and that these are often localized to the head^14^. The second component (*Stomach Activation*) mainly reflected activation in the stomach, and was primarily represented in disgust; this component may thus reflect visceral activity, including sensations from the gut^50^. The third component (*Legs Deactivation)* reflected contributions from negative, distress-related emotions such as sadness, fear and shame, as well as disgust. As this component represented deactivation (i.e., the sensation of the body getting weaker or heavier), this may reflect aspects of the freezing response to stress, which has been suggested to be implicated in disorders such as depression^51^. Interestingly, the *Stomach Activation* component also loaded strongly on these emotions, perhaps reflecting alterations in digestion and gastric signalling which occur during stress^52^. Finally, the fourth component (*Heart Activation*) was present particularly in high-arousal positive emotions such as happiness and pride, as well as surprise. Thus, this component likely represents perceived heart rate changes associated with the physiological reactions elicited by these emotions^2^.

The finding that the amount of colouring in the heart and stomach in body maps of emotion was negatively correlated with the *Antisocial Attitudes* personality component suggests that somatosensory representations of emotion, particularly those associated with physiological responses, are reduced in individuals with antisocial tendencies. This finding is consistent with a considerable body of previous work implicating somatosensory processes in social and empathic perception. For instance, TMS studies have found that inhibition of somatosensory cortex reduces participants’ skill at judging emotional expressions and performance on an affective go/no-go task^11^, and imaging and lesion studies have implicated somatosensory cortex in the perception of emotional facial expressions^10,53,54^, as well as in empathy for pain^55^.

Our result showing that emotional somatosensation from the heart is important for social perception and prosocial behaviour is also in agreement with a large body of literature on the importance of cardiac interoception in emotion processing. As changes in heart rate accompany changes in arousal and stress, the heart is one of the most commonly implicated structures in emotion experience^2^. Moreover, cortical processing of heartbeat signals has been associated with first vs. third-person perspective taking, a process which underlies theory of mind and thus empathic understanding^56^. For instance, neural processing of heartbeats has been shown to be increased during an empathy task in which participants made affective or physical judgements of facial expressions^57^. Our results support the growing view that cardiac interoception is a key substrate of empathic individuals’ ability to understand their own and others’ emotions. Similar interpretations apply for somatosensation from the stomach, as digestion and gastric signals are also affected by emotions^58^. Moreover, gastric signals have likewise been proposed to underlie first-person perspective and thus, indirectly, the ability to mentalize and understand the perspectives of others^59^.

A number of theories of psychopathy implicate blunted emotional responses in the disorder, providing an interesting context to our findings. For example, the low-fear model of Lykken^60^ proposes that psychopaths have reduced fear responses to aversive stimuli, preventing moral conditioning, while the Violence Inhibition Model and Integrated Emotion Systems models^61,62^ propose deficits in amygdala functioning as key to the condition. Our findings showing reduced emotional somatosensation in individuals high in antisocial personality traits support these theories, and point to altered emotional somatosensation as a potential mediating mechanism between neurobiological alterations in psychopathy and the experience of blunted affect. Furthermore, our findings extend these theories and the relevance of altered emotional experience to non-clinical antisocial traits, such as non-clinical psychopathy, low agreeableness, and social dominance.

Interestingly, the *Head* Activation BSM component correlated negatively with *Impulsivity*. As discussed above, this head activation component may be associated with the somatic experience of cognition, following its correlation with mental salience and in accordance with recent work using the BSM paradigm^14^. Physiologically, this may be related to muscle activation: for example, cognitive effort is associated with activation of the frontalis muscle in the forehead, which may be a reflection of the common experience of “furrowing one’s brow” when deep in thought about a difficult subject^63^. Thus, despite the common idiomatic expression that impulsive people are “hot-headed”, our results suggest that these cognitive feelings are reduced in these individuals, which may contribute to the reduced influence of top-down cognitive control during emotion experience in impulsive individuals^64^. However, the directionality of this effect is unclear – while somatosensory representations of emotion may influence personality development, it is equally possible that reduced top-down control in impulsive individuals extends to the somatosensory experience of emotion as well.

Together, our findings suggest that dimensions of emotional somatosensation, which cut across individual emotions, are relevant for personality. While bodily maps of emotion have been discussed previously as categorically distinct, evolutionarily-determined physiological signatures of basic emotions^12,15^, our results here suggest a more dimensional approach. Most of the principal components we observed were represented strongly in multiple emotions, rather than being specific to any given one. Moreover, our findings considering the distance between BSMs (see below), revealed that the group-average maps of emotion are substantially more similar than any individual subject’s maps. That is not to say that the BSMs for the different emotions were indistinguishable from each other, as the classifier was able to accurately classify the body sensation maps well above chance. However, instead of the current interpretation that the accurate classification of BSMs reflects emotion-specific, categorically distinct maps, our results point to a circumplex model of emotional somatosensation: in this model, sensations for different emotions are described by the relative weights of underlying components, such as the heart, head, and stomach activation components observed in the present study (see Clark-Polner et al.^65^ for a similar argument regarding classifiers and basic emotions).

### Emotional granularity and body sensation maps: the importance of representative and accurate somatosensation

Our second major set of findings concerned emotional granularity, as measured by the similarity of bodily maps across emotions and participants. Emotion differentiation was previously assessed by Torregrossa et al.^17^ and Palser et al.^16^ using the classifier approach described above to argue that BSMs were less differentiated in autism and schizophrenia patients with respect to healthy controls. Following this work, we trained a classifier on our BSM data, taking its classification confidence as a measure of emotion differentiation. We followed up this analysis by calculating participants’ emotion differentiation and representativeness (indexed by cosine distance-based metrics), to determine the driving factors underlying accurate classification of emotions. We found that interoception was positively associated with classification confidence, while antisocial attitudes and negative affect were negatively correlated. In the case of antisocial attitudes, the lower classification confidence occurred despite the concomitant positive correlation with intra-individual, inter-emotion distance. These counterintuitive findings can be explained by the fact that BSM representativeness (distance to the group mean) and differentiation (within-subject distance between emotions) were in fact negatively correlated (r = − 0.76). Indeed, the group-average templates exhibited considerably higher similarity between emotions (average cosine similarity = 0.77) than most subjects’ individual BSMs (average cosine similarity = 0.17). As mentioned in the “Methods” section, successful emotion classification requires both high inter-class and low intra-class variability^47,48^. Results from the classification analysis suggest that the higher inter-emotion BSM differentiation in participants with high antisocial traits was not sufficient to confidently categorize them accurately, given their higher dissimilarity to the group averages. Consistent with this, we observed that the correlation between *Antisocial Attitudes* scores and emotion differentiation was in fact mediated by its relation with representativeness (Sobel test; *p* = 0.04).

Emotional granularity reflects the experience of fine-grained distinctions in emotion^23^ and is an important aspect of individual variation in emotion experience. That is, it has a wide variety of associations with personality and life outcomes^66^. Methods of assessing emotional granularity typically involve correlating instances of emotion labels collected over experience sampling, with the rationale that a lower correlation implies a more differentiated experience of emotion^67^. However, the bodily maps of emotion paradigm has the key advantage of producing a multidimensional representation of an individual’s actual emotional experience, allowing for the direct and quantitative measurement of emotion differentiation. Indeed, previous studies have used classification accuracy as a proxy for emotion differentiation, showing that emotions are less differentiated in schizophrenia^17^. Likewise, in our data classification confidence was related to several personality factors previously associated with emotional granularity, including negative affect^68^, interoception^22^, and prosocial tendencies such as empathy^21^. Thus, the BSM paradigm allows for a conceptually different, complementary assessment of emotional granularity, focusing on the similarities and differences between the experience of different emotions, in contrast with traditional methods that instead assess the temporal coherence between the occurrence of different emotions.

Classifiers such as the linear discriminant analysis used in our study attempt to maximize the ratio of between-class variance to within-class variance^47^. Thus, associations such as the ones we found between classification confidence and personality may be driven either by differences in the degree of intra-individual separation between classes, or in the degree of inter-individual homogeneity within classes. In our study, we found that the correlation of empathy with classifier confidence was driven primarily by its strong association with the representativeness of participants’ maps; that is, how much any given map resembled the group average. Our results thus suggest that within-category homogeneity across individuals is a key factor in determining associations with personality, with between-category variability less so, at least the personality and granularity measures considered here. This result carries implications for research on emotional granularity, where the aspect of within-category, between-subject homogeneity is rarely considered. Indeed, it suggests that some of the proposed benefits of emotional granularity and observed relationships with personality (such as its relationship with empathy) may stem from the accuracy or representativeness of an emotional experience, and not just (or mainly) from its distinctiveness relative to the experience of other emotions. Interestingly, however, negative affect correlated significantly with emotion differentiation, but not with any of the BSM PCA components or with our measure of representativeness: this suggests that there may be different physiological routes to undifferentiated emotion experience. Further research is needed to investigate this possibility, as well as to apply our design to other factors associated with emotional granularity such as resilience and psychosocial functioning^66^.

Interestingly, the finding that antisocial attitudes negatively correlate with BSM representativeness suggests that another key feature of this personality trait may be that it places the individual in the margins of the distribution of emotional somatosensation among the population, thus reducing their ability to recognize and interpret other people’s emotions. According to the Perception–Action Model of empathy^69^, observation of the emotional state of another activates corresponding emotional states in oneself, including their somatosensory component. If there are individual differences in emotional somatosensation, then the vicarious emotional representation in the self may be more or less similar to the other’s. Thus, if antisocial individuals’ emotional somatosensation is idiosyncratic, this may reduce the likelihood of affective empathy with other individuals’ expressions, by virtue of the reduced overlap between their respective somatosensory representation of emotion. Consistent with this hypothesis, several studies have shown that psychopaths show reduced somatosensory and motor response when viewing expressions of pain or emotion in others^70–72^; but see^73^. Our findings also agree with previous findings by Sachs et al. that empathy was predictive of self-other overlap in BSMs^74^; indeed, if high-empathy individuals are closer to the group-average BSMs, they will likely overlap more with a larger set of others’ maps.

### Processes underlying BSM generation: somatosensation, appraisal, and categorization

In order for the relationships observed between participants’ BSMs and personality to be interpretable, a theoretical model of the processes underlying the BSM task is informative and necessary. Recently, an integrative model of physiological, appraisal, and cognitive factors in emotion has been proposed^75^, which can provide a useful framework within which to interpret our findings. Following this model, we propose that there are 4 stages involved in the production of a participant’s bodily sensation map where individual variation might have an effect on the BSMs. First, individual differences in the actual emotion-related physiological response: for example, a participant who is more physiologically reactive to threats^76^ and has a larger heartrate increase in response to fearful situations may experience greater changes in emotional somatosensation, and thus colour more intensely on the BSM task. Second, variation in an individual’s sensitivity to emotional somatosensation: if an individual is more aware of their somatic sensations, they will be more able to report them in the body maps task. Third, variation in an individual’s conceptual ability to associate somatosensation with emotion: for example, if an individual experiences sensation from their heart whenever they feel fear, but interpret it as symptoms of a cardiac abnormality (as in somatization:^77^) they will not report sensation from the heart in the fear BSM. Finally, memory retrieval processes may also affect the drawing of the maps: a participant’s recollection of their emotional experience may influence the intensity or specificity of the maps.

Existing data suggests that the second variable listed above (variation in sensitivity or attention to emotional somatosensation) produces the largest contribution to inter-individual variation in BSMs. The original study by Nummenmaa et al. found that BSMs were highly similar between different modes of evoking emotion, including emotion labels, emotion-inducing videos, and asking participants to colour BSMs for other individuals based on emotional facial expressions. This suggests that variation at cognitive categorization and memory levels may be small, though BSM similarity between modalities was only assessed at the group level, and not at the individual level (i.e., within-individual similarity of BSMs across the different evoking modes). In contrast, a previous study^78^ found that interoceptive accuracy, using a heartbeat detection task, was predictive of magnitude and specificity of BSM colouration, suggesting that there may be significant variation at the level of individual sensitivity to somatosensation; our data support this, finding correlations between BSM factors and interoceptive sensitivity. Nonetheless, future work should confirm the relationships found here using different modes of evoking the emotions for each BSM, and by adding other, more objective measures, for example using physiological recordings or neuroimaging techniques.

### Limitations and future directions

While the present work addresses the main questions raised in the introduction, there are a number of issues that may limit the generalizability of our findings. The majority of our participants were university students taking psychology courses, thus likely of a medium–high socioeconomic status, and thus not representative of the general population on several psychological dimensions^79^. While corrected for multiple comparisons, our* p* values also were fairly high. Thus, the results reported here may be considered somewhat preliminary, and, as with any initial set of findings, there is a need for replication, especially using broader samples^80^. Moreover, our sample was composed mostly of (self-identified) women (as do most studies recruiting volunteer healthy participants), leaving open the question of the generalizability of our findings to men and non-binary individuals. However, we note that previous research using the bodily maps of emotion paradigm has found that BSMs are largely consistent across cultures and sexes^15^, suggesting that these factors may not substantially affect our results. Nonetheless, further studies designed to directly test this should be carried out.

The present work demonstrates associations between dimensions of personality and emotional somatosensation. However, given the a cross-sectional and correlational nature of our procedure and analysis, respectively, we are unable to establish causal relationships between personality and somatosensory processes. Future studies are necessary to determine this, as well as which of the different factors underlying emotional somatosensation, as described above, play a role in this relationship.

While the bodily maps of emotion task is useful for determining the location of emotion-related somatosensation, a key limitation of the method is that the quality of emotional somatosensation is not well-specified: the task asks participants simply to paint “activation” or “deactivation”, and typically these labels are treated as two poles of a single activation-deactivation continuum. However, somatosensation for different emotions may have distinct phenomenology—for example, the tightness in the chest felt for anxiety is not the same sensation as the full, brimming sensation felt in happiness or pride, yet both may be coloured as “activation” in the bodily maps procedure. Thus, future work should expand the bodily maps of emotion paradigm by incorporating different qualities of somatosensation.

## Conclusion

Patterns of body sensation are crucial for the experience of emotion, but individual differences in these sensations have rarely been studied in the context of personality. Using a behavioural paradigm in which participants “paint” areas of their body where they feel sensation during different emotions, we investigated the relationship between people’s bodily sensations of emotion and their personality using data-driven principal components regression. Several aspects of participants’ emotion-related body sensations were related to antisocial tendencies and impulsivity, including activation in the heart, viscera, and head. Furthermore, classification accuracy was related to antisocial attitudes and, to a lesser degree, interoception, an effect which was mainly driven by the category-representativeness of BSMs, rather than intra-individual emotional differentiation. These results suggest that while emotions have generally consistent somatosensory fingerprints, individuals may be more or less sensitive to different aspects of these sensations, and that these differing somatosensory representations of emotion may be related to specific aspects of personality.

## Supplementary Information

Supplementary Information.

## Data Availability

The data from the above study is available from the corresponding author upon reasonable request.
