# Supplementary material for: Antisocial and impulsive personality traits are linked to individual differences in somatosensory maps of emotion
Source: Sci Rep. 2023 Jan 12;13:675. doi: 10.1038/s41598-023-27880-1 (PMC9837047; doi:10.1038/s41598-023-27880-1)
Supplement: Supplementary file 1 — Supplementary Information. [file 41598_2023_27880_MOESM1_ESM.docx]

**Supplementary materials**

*Full loadings for the PCA decompositions*


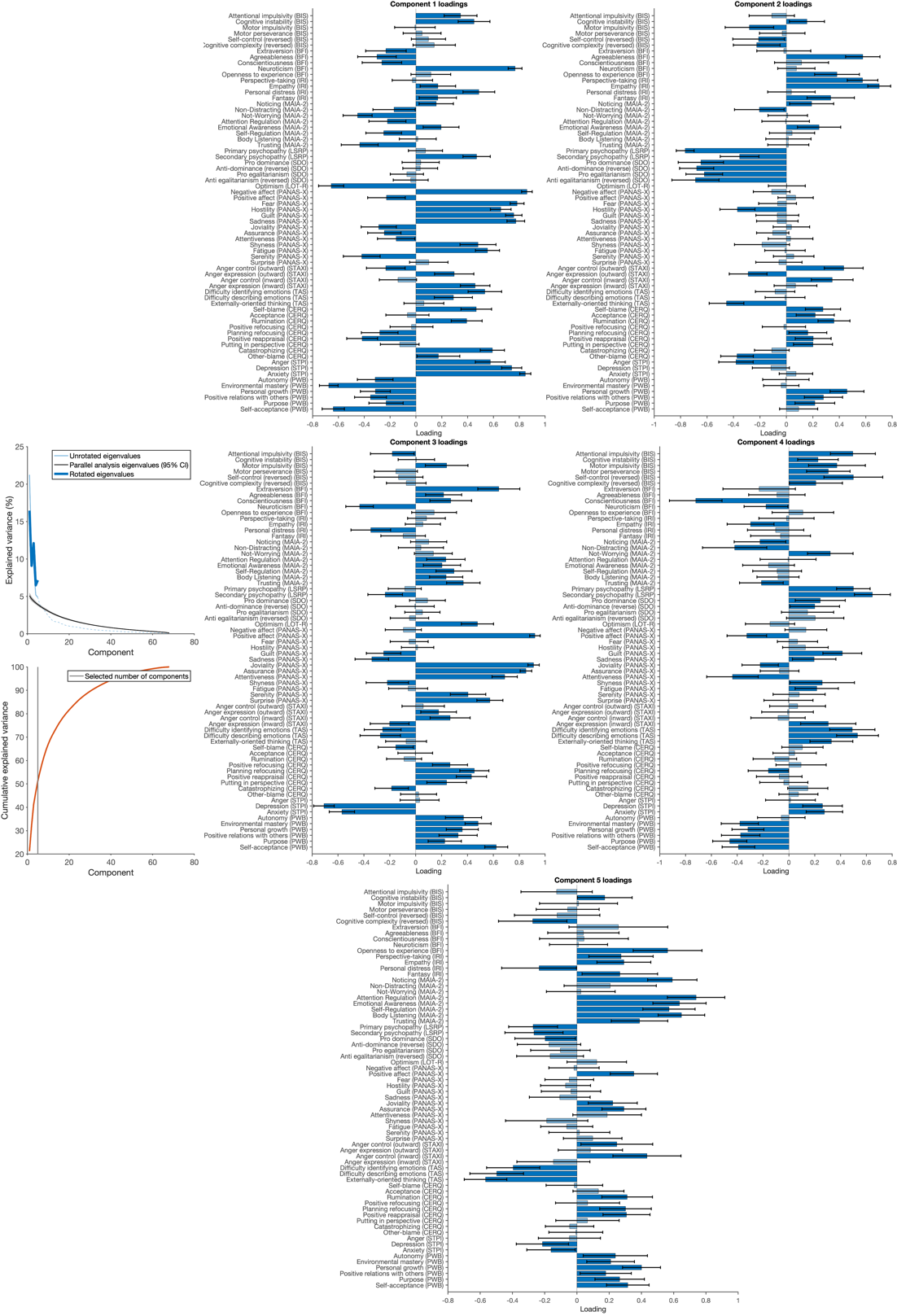


**Figure S1.** Full loadings for the personality PCA decomposition. Left: Eigenvalue plots as in figure 2. Bold blue line shows the eigenvalues of the rotated components. Light blue line shows the eigenvalues of the unrotated components; the dashed part reflects eigenvalues not significant following the permutation test. Black line shows the eigenvalues from the permutation test and the 95% confidence interval of these (grey shading). Right: loadings for each component on all personality scales. Bars represent loadings, error bars reflect bootstrap confidence intervals. Bars are shaded dark blue if they are significant (bootstrap CI excludes 0), light blue if they are not significant.
